# Metabolomic profiling of rectal microorganisms in Tibetan sheep across cold and warm seasons

**DOI:** 10.3389/fvets.2025.1513571

**Published:** 2025-03-21

**Authors:** Qi-Tala An, Zhipeng Zhao, Yaxiong Ren, Xia Liu, Liangwei Yao, Siyuan Chen, Zhikuan Yuan, Peijian Feng, Wenhao Li, Xiaohua Du

**Affiliations:** ^1^College of Veterinary Medicine, Gansu Agricultural University, Lanzhou, China; ^2^College of Animal Science and Veterinary Science, Qinghai University, Xining, China

**Keywords:** Tibetan sheep, metabolomics, season, nutrition metabolism, rectal microbiology

## Abstract

The intestinal metabolites of Tibetan sheep play a vital role in the integrated regulation of the host and the microbe-gut-brain axis. The current study sought to investigate the characteristics of alterations in rectal metabolites and their functional implications during the cold and warm seasons in Tibetan sheep. A cohort of 12 ewes, approximately 1 week ± 1 month in age, exhibiting good body condition and a similar genetic background, was selected for metabolomic analysis of rectal contents collected during the warm season (August) and the cold season (December). The findings revealed significant differences in the rectal microbial metabolites of Tibetan sheep between the two seasons (*P* < 0.05), with a total of 476 differential metabolites identified in the positive ion mode (148 up-regulated and 328 down-regulated) and 383 differential metabolites in the negative ion mode (135 up-regulated and 248 down-regulated). These differential metabolites were mapped to 12 KEGG metabolic pathways (*P <* 0.05), including fatty acid biosynthesis, arachidonic acid metabolism, secondary bile acid biosynthesis, propionic acid metabolism, lysine degradation, and arginine and proline metabolism, which are linked to lipid metabolism, carbohydrate metabolism, and amino acid metabolism, respectively. The content of deoxycholic acid in the intestinal tract of Tibetan sheep was significantly higher during the cold season compared to the warm season (*P <* 0.05), while propionic acid was significantly lower (*P <* 0.05). These metabolites are involved in secondary bile acid biosynthesis and propanoate metabolism pathways. These results indicate significant seasonal variations in rectal microbial metabolites in Tibetan sheep. The identified metabolites may play a crucial role in regulating energy metabolism, inflammatory responses, and immune functions, thereby enhancing the adaptability of Tibetan sheep to the challenges posed by cold-season conditions.

## 1 Introduction

Tibetan sheep are recognized as one of the three principal breeds of rough wool sheep in China, predominantly found in the Qinghai-Tibetan Plateau and adjacent regions such as Sichuan, Yunnan, and Gansu. With an estimated population of approximately 33 million, Tibetan sheep represent the largest sheep population in China and are a crucial genetic resource for livestock in the Qinghai-Tibetan Plateau. This breed has undergone extensive evolution through both natural and artificial selection, leading to distinctive adaptations to the challenging alpine and low-oxygen conditions of the Tibetan Plateau. Key attributes of Tibetan sheep include their resistance to hypoxia and cold temperatures, their ability to thrive on coarse forage, and their robust disease resistance (1). The growth cycle of these grasses can be divided into three distinct phases based on climatic variations: the greening period (April to June), the peak growth period (June to October), and the drying period (October to April). The greening and drying periods correspond approximately to the warm and cold seasons, respectively. Research indicates that the crude protein (CP) and ether extract (EE) contents of the pasture are significantly higher during the greening stage than during both the peak growth and drying stage (2). Conversely, the acid detergent fiber (ADF) and neutral detergent fiber (NDF) contents are higher during the drying stage than the greening stage. Under natural grazing conditions, the dry matter intake of Tibetan sheep is recorded at 116 g/kgBW^0.75 during the warm season, which decreases to 59 g/kgBW^0.75 in the cold season. The nutrient deficiencies in forage grasses during the drying period impede their capability to meet the nutritional requirements of Tibetan sheep, thereby presenting considerable challenges to the development of the Tibetan sheep industry.

The gastrointestinal tract of ruminants hosts a significant diversity of microorganisms. Under typical conditions, these microorganisms have adapted to the host’s living environment, establishing an ecological equilibrium among the microorganisms, the host, and the surrounding environment, which is essential for optimal gut function (3). It is widely acknowledged that factors such as diet composition, environmental conditions, age, and genotype are the primary determinants of gut microbial diversity in ruminants (4, 5), with diet composition being the most significant external influence (6). Variations in the nutrient composition of pasture grasses between the cold and warm seasons suggest that seasonal environmental changes may influence the composition of gut microorganisms in Tibetan sheep. Roughage rich in cellulose and hemicellulose can promote the growth of fiber-decomposing bacteria in the rumen of ruminants, such as Ruminococcus and Fibrobacter. These bacteria can effectively degrade cellulose and produce volatile fatty acids, providing energy for ruminants. In contrast, high-concentrate feeds rich in starch and protein promote the growth of amylolytic rumen bacteria, such as Prevotella, and Clostridia, which enhance the digestion of starch and proteinmore efficiently (7).

The “microbiota-gut-brain axis” refers to a bidirectional communication network that links brain and gastrointestinal functions through the central nervous system and various enteric nervous systems. This network is primarily composed of neurons, endocrine signals, and immune factors. It has been proposed mechanism indicates that metabolites generated by gut microbiota can influence the central nervous system via multiple pathways, while the central nervous system can, in turn, modulates the gut microbiota composition (8). The functionality of gut microbiota in ruminants can be evaluated through the examination of gut microbial metabolites (9), which are integral to the collective regulation of the host and the brain-gut axis. The rectum is the end of the digestive tract, closer to the outside of the body, and it is relatively easy to collect the contents of the rectum, which is less difficult and risky to operate on and causes less damage to the body. At the same time, the contents of the rectum contain the “final form” of digestive metabolites from each segment of the intestine after it has gone through the digestion and absorption process of the entire digestive tract, which can comprehensively reflect the metabolic situation of the entire intestinal tract. However, limited research has investigated how the gut microbiota metabolome varies seasonally in Tibetan sheep. Therefore, the current study aims to explore the metabolomic profiles of intestinal microorganisms in Tibetan sheep during both cold and warm seasons. This research intends to elucidate the characteristics of intestinal metabolites and their functional variations across the two seasons, as well as to clarify the associated functions linked to their metabolic pathways. Ultimately, the study aims to provide insights into the bidirectional regulation of the brain-gut axis and how seasonal differences affect nutrient metabolism in Tibetan sheep.

## 2 Materials and methods

### 2.1 Experimental materials

The animal experiment protocols involved in this study were all approved by the Animal Experiment Ethics Committee of Gansu Agricultural University (No. GSAU-Eth-VMC-2023-036).

Tibetan sheep were sourced from a single herd situated in Qilian County, Qinghai Province. A total of 12 ewes, approximately 1 month old ± 1 week, were selected based on their robust health and comparable genetic backgrounds. These ewes were ear-tagged, numbered, and incorporated into the existing flock. The one-year-old female Tibetan sheep had reached a certain stage of physiological development, and the gut microbiota has also become relatively stable. Due to the regularity of the estrous cycle and the absence of factors that may affect hormone levels, such as competition for mates seen in male sheep, the experimental data were considered more accurate and convincing. The management of the sheep adhered to a traditional natural grazing system, with no supplemental feeding provided during the grazing period. The animals had unrestricted access to both feed and water. The primary types, compositions, and nutritional contents of the pasture grasses during the cold and warm seasons were documented in the literature by Fan et al. (10). Slaughtering and sample collection were conducted during the warm season (August 2023) and the cold season (December 2023), respectively. Prior to slaughter, all Tibetan sheep underwent a fasting period of 16 h. Fresh fecal samples were collected rectally, immediately placed in a prepared liquid nitrogen tank, and transported to the laboratory, where they were stored at −80°C for subsequent metabolomics analyses.

### 2.2 Sample preparation for liquid chromatography-mass spectrometry analysis

Rectal fecal samples were gradually thawed at 4°C. A suitable volume of the samples was then combined with a pre-cooled solution of methanol/acetonitrile (Merck, 1,499,230–935) and water in a 2:2:1 (v/v) ratio. The mixture was vortexed thoroughly and subjected to low-temperature ultrasoni treatment for 30 min. Following this, the samples were centrifuged in a high-speed centrifuge (Eppendorf 5430R) for 20 min at 4°C and 14,000 g. The resulting supernatant was vacuum-dried to remove solvents. For mass spectrometry analysis, 100 μL of aqueous acetonitrile (in a 1:1 v/v ratio with water) was added to the dried supernatant, which was then vortexed and centrifuged at 14,000 g for 15 min at 4°C. The supernatant obtained from this process was utilized for subsequent online analysis.

Quality control (QC) samples were prepared by combining equal volumes of the samples under investigation. These QC samples serve multiple functions: they are used to assess instrument performance and to calibrate the chromatography-mass spectrometry system prior to injection. Additionally, QC samples are utilized to evaluate the stability of the system throughout the duration of the experiment.

### 2.3 Onboard testing

Chromatographic analysis was performed using an Agilent 1,290 Infinity LC ultra-high performance liquid chromatography (UHPLC) system, which was equipped with a hydrophilic interaction liquid chromatography (HILIC) column. The column temperature was maintained at 25°C, with a flow rate of 0.5 mL/min and an injection volume of 2 μL. The mobile phases employed were as follows: Phase A consisted was an aqueous solution of 25 mM ammonium acetate and 25 mM ammonia, while Phase B was acetonitrile. The gradient elution protocol was executed as follows: from 0 to 0.5 min, 95% of Phase B was used; from 0.5 to 7 min, Phase B was linearly reduced from 95 to 65%; from 7 to 8 min, Phase B was further decreased linearly from 65 to 40%; from 8 to 9 min, Phase B was held constant at 40%; from 9 to 9.1 min, Phase B was linearly increased from 40 to 95%; and finally, from 9.1 to 12 min, Phase B was maintained at 95%. During the analysis, samples were stored in the autosampler at a temperature of 4°C. To minimize the effects of fluctuations in the instrumental detection signal, samples were analyzed in a randomized order. QC samples were included in the sample queue to monitor instrument stability and ensure data reliability.

Mass spectrometry analyses were conducted using an AB Triple TOF 6600 mass spectrometer, which capture both primary and secondary spectra.

The electrospray ionization (ESI) source conditions following hydrophilic interaction liquid chromatography (HILIC) separation were as follows: Ion Source Gas 1 (Gas 1): 60, Ion Source Gas 2. The source temperature was established at 600°C. The Ion Spray Voltage Floating (ISVF) was adjusted to ±5,500 V for both positive and negative modes. The time-of-flight mass spectrometry (TOF MS) scan was conducted over a mass-to-charge (m/z) range of 60–1,000 Da, while the product ion scan was performed within an m/z range of 25–1,000 Da. The accumulation time was 0.20 s per spectrum for the TOF MS scan, and 0.05 s per spectrum for the product ion scan.

Secondary spectra were obtained using information-dependent acquisition (IDA) in high sensitivity mode. The declustering potential (DP) was set to ±60 V for both positive and negative modes, and the collision energy was maintained at 35 ± 15 eV. The IDA settings included the exclusion of isotopes within 4 Da, with a maximum of 10 candidate ions were monitored per cycle.

### 2.4 Data processing

The raw data were converted into. MzML format using ProteoWizard, after which the XCMS software was used for peak alignment, retention time correction, and peak area extraction. The parameters for the XCMS analysis were set as follows: for peak picking, the centWave m/z tolerance was set to 10 ppm, the peak width was defined as c(10, 60), and the prefilter was set to c(10, 100). In terms of peak grouping, the parameters included a bandwidth (bw) of 5, an m/z width (mzwid) of 0.025, and a minimum fraction (minfrac) of 0.5. Subsequent to the data extraction via XCMS, the dataset was initially evaluated for completeness. Metabolites with more than 50% missing values within a group were excluded from further analysis. Missing values were addressed through K-nearest neighbors (KNN) imputation, extreme values were removed, and total peak area normalization was performed to ensure comparability across samples and metabolites.

## 3 Results

### 3.1 Orthogonal-partial least squares discriminant analysis

**Figure 1:**
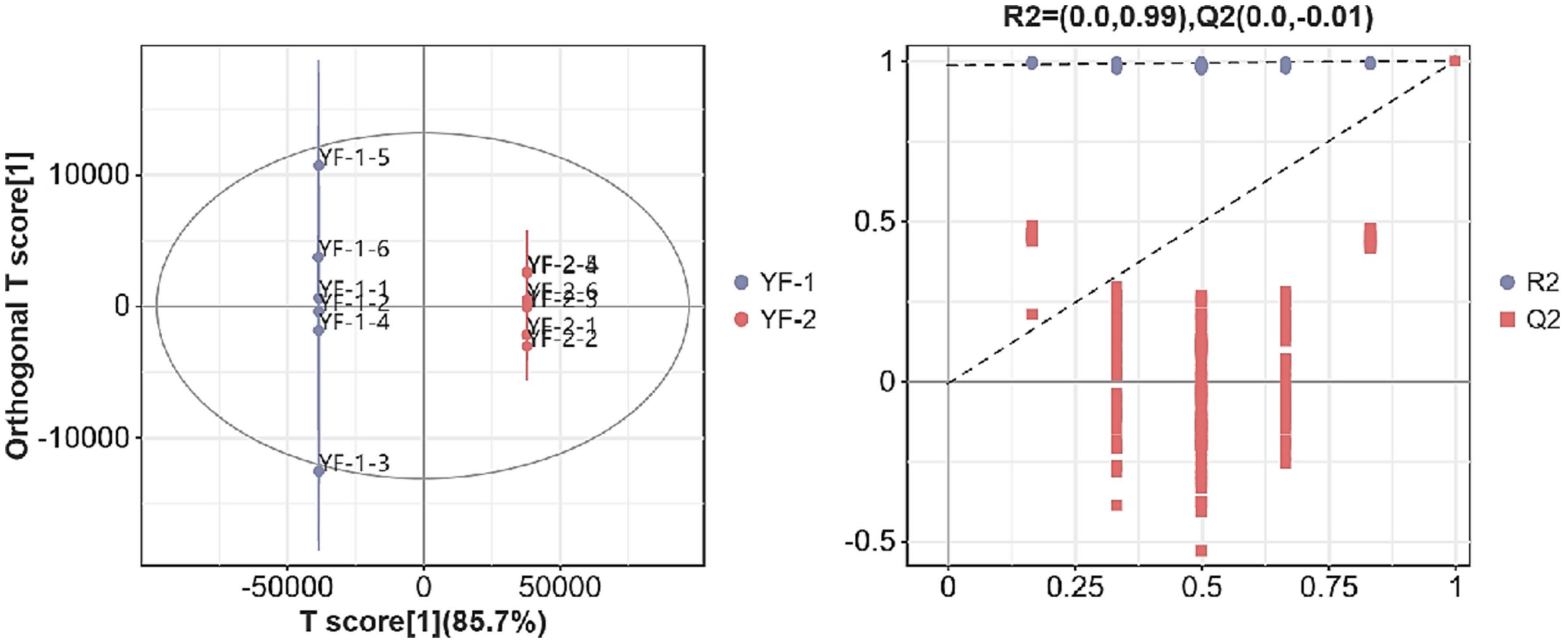
Plot of OPLS-DA scores and substitution test in the positive ion mode.

**Figure 2:**
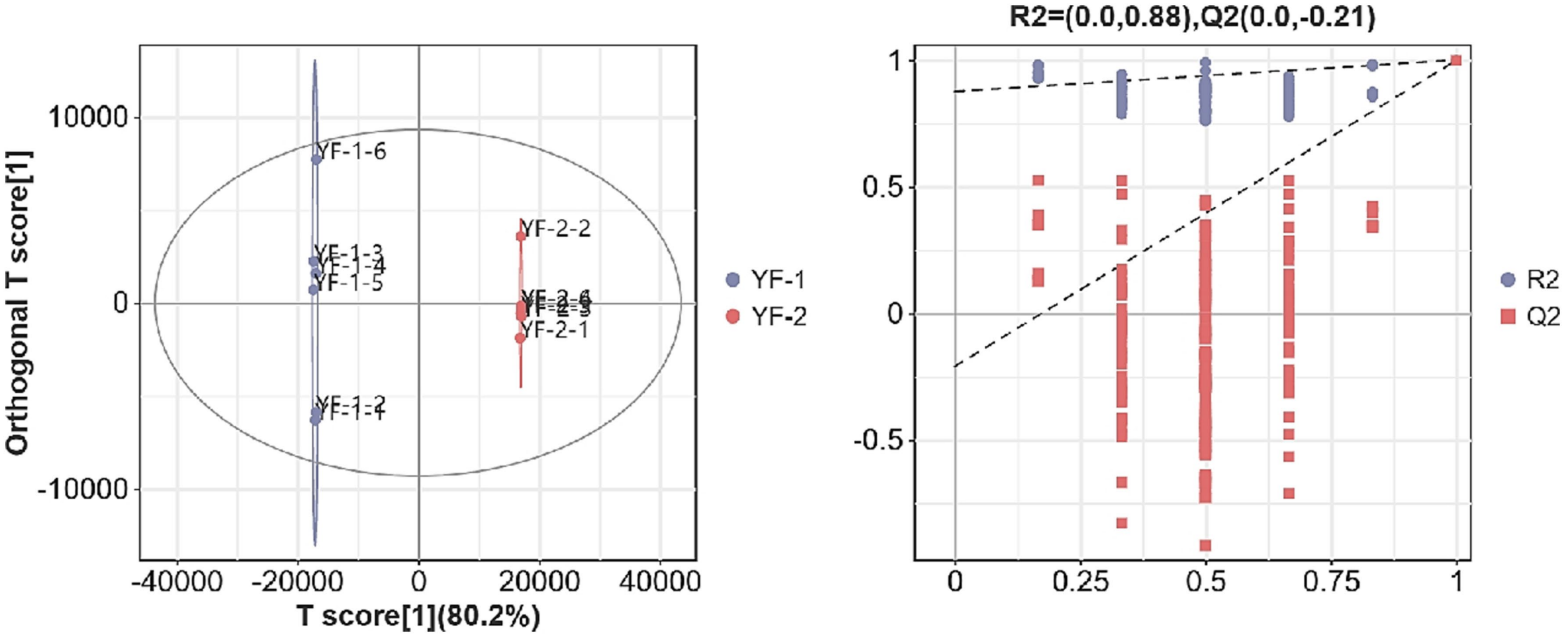
Plot of OPLS-DA scores and substitution test in negative ion mode.

As shown in Figures 1, 2, the intestinal metabolite profiles from warm-season Tibetan sheep and cold-season Tibetan sheep displayed notably different confidence intervals. Intra-group variation was minimal, while inter-group differences were pronounced. Additionally, all *R*^2^-values approached 1, with *R*^2^-values surpassing Q^2^, and the intercept of the Q^2^ regression line with the Y-axis was found to be less than 0. These results suggest that the model used in this study effectively characterizes the samples, confirming the accuracy and reliability of the data for subsequent analyses.

### 3.2 Basic analysis of rectal metabolite differences

**Figure 3:**
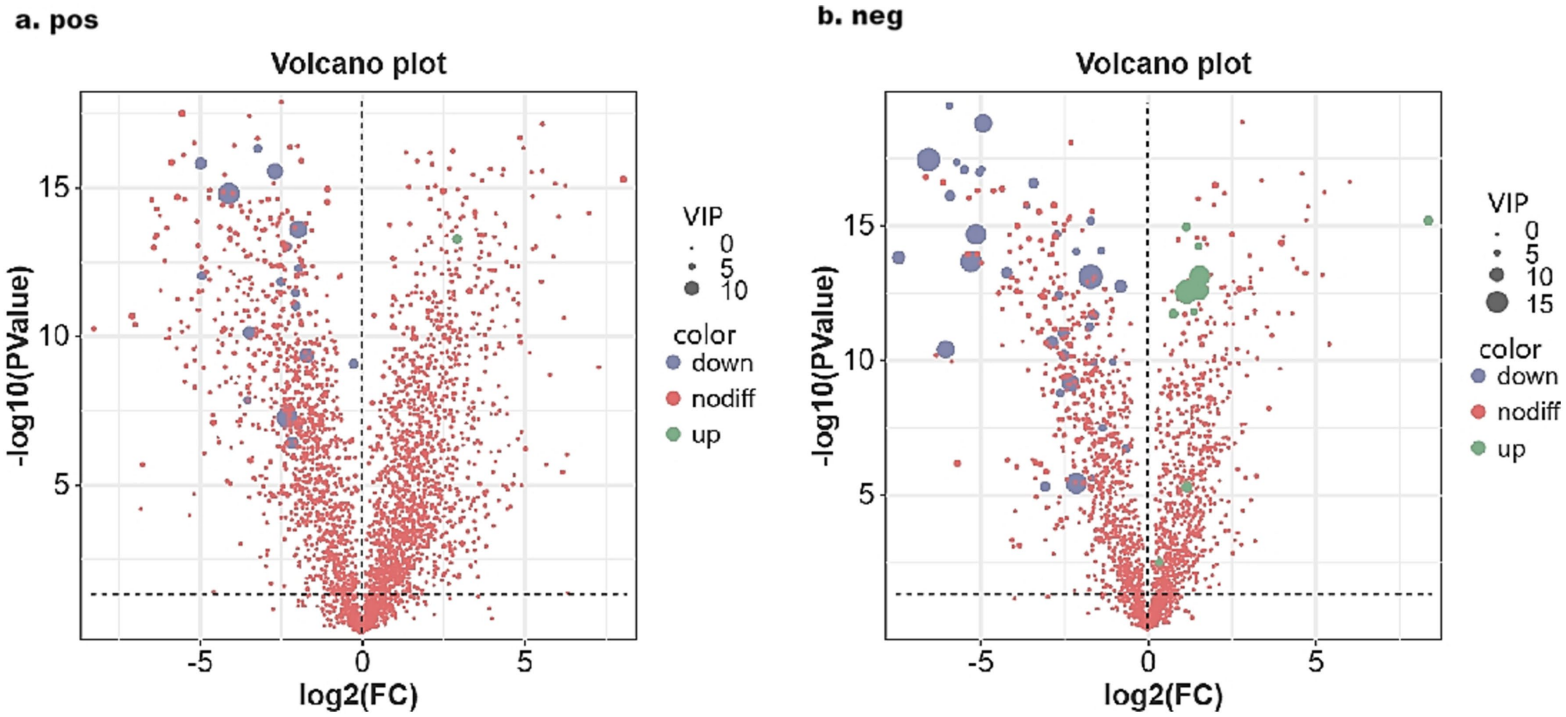
Volcanic map of rectal differential metabolites in Tibetan sheep in warm and cold seasons under positive **(A)** and negative **(B)** ion modes.

**Figure 4:**
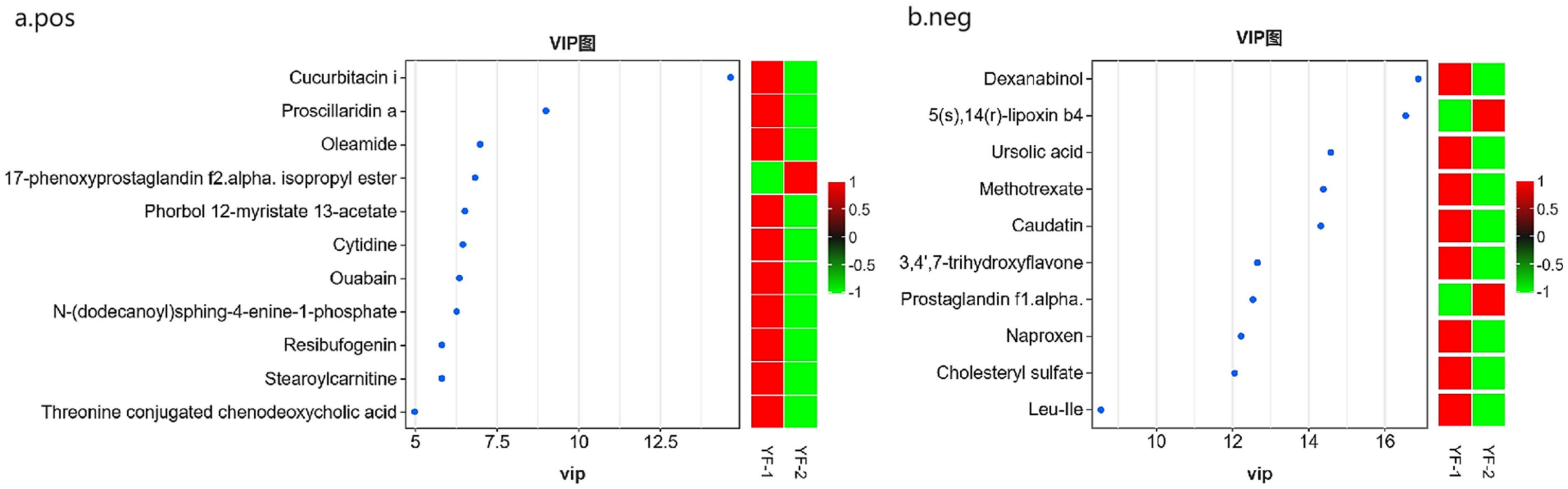
VIP maps of rectal differential metabolites in Tibetan sheep in warm and cold seasons under positive **(A)** and negative **(B)** ion mode..

Using Orthogonal Partial Least Squares Discriminant Analysis (OPLS-DA) alongside a Variable Importance in Projection (VIP) threshold greater than 3, a *p*-value of less than 0.05 from univariate analysis via the *T*-test, and a log_2_ fold change (| log_2_ |FC) of greater than or equal to 1 or less than or equal to −1, a comprehensive analysis of all metabolites detected in both positive and negative ion modes was conducted. Volcano plots and VIP plots were used for visual representation (see Figures 3, 4). The rectal fecal samples from the warm-season and the cold-season Tibetan sheep groups yielded 476 and 383 differential metabolites, respectively, across both ion modes. In the positive ion mode (see Figure 3A), 148 metabolites were up-regulated, while 328 were down-regulated. In the negative ion mode (see Figure 3B), 135 were up-regulated and 248 were down-regulated. A selection of these differential metabolites is detailed in Table 1. Notably, the primary metabolites that were up-regulated in the positive ion mode (see Figure 4A) included Flecainide, Prolyl-Tryptophan, and Glycoursodeoxycholic acid, whereas the down-regulated metabolites included Leucyl-leucine, Cytidine, and Stearoylcarnitine. In the negative ion mode (see Figure 4B), the up-regulated metabolites predominantly consisted of Glutamylglycine, Prostaglandin F1α, and 5(S),14(R)-lipoxin B4, while the down-regulated metabolites primarily included Prolyl-Threonine, Ursolic acid, and Methotrexate.

**Table 1:** Selected differential metabolites in the rectum of Tibetan sheep during the warm and cold seasons in positive and negative ion modes.

Metabolite chemical name	VIP value	log2(FC)	*P*-value
Dexanabinol	16.872	−1.693	8.46E-14
5(s),14(r)-lipoxin b4	16.541	1.179	3.05E-13
Cucurbitacin I	14.664	−2.291	5.72E-08
Ursolic acid	14.576	−5.265	2.31E-14
Methotrexate	14.385	−2.121	3.89E-06
Caudatin	14.315	−5.115	2.27E-15
3,4′,7-trihydroxyflavone	12.653	−4.908	1.72E-19
Prostaglandin f1.alpha.	12.534	1.555	2.80E-13
Naproxen	12.222	−6.019	4.18E-11
Cholesteryl sulfate	12.053	−2.306	7.25E-10

### 3.3 Differential metabolite KEGG enrichment analysis

**Figure 5:**
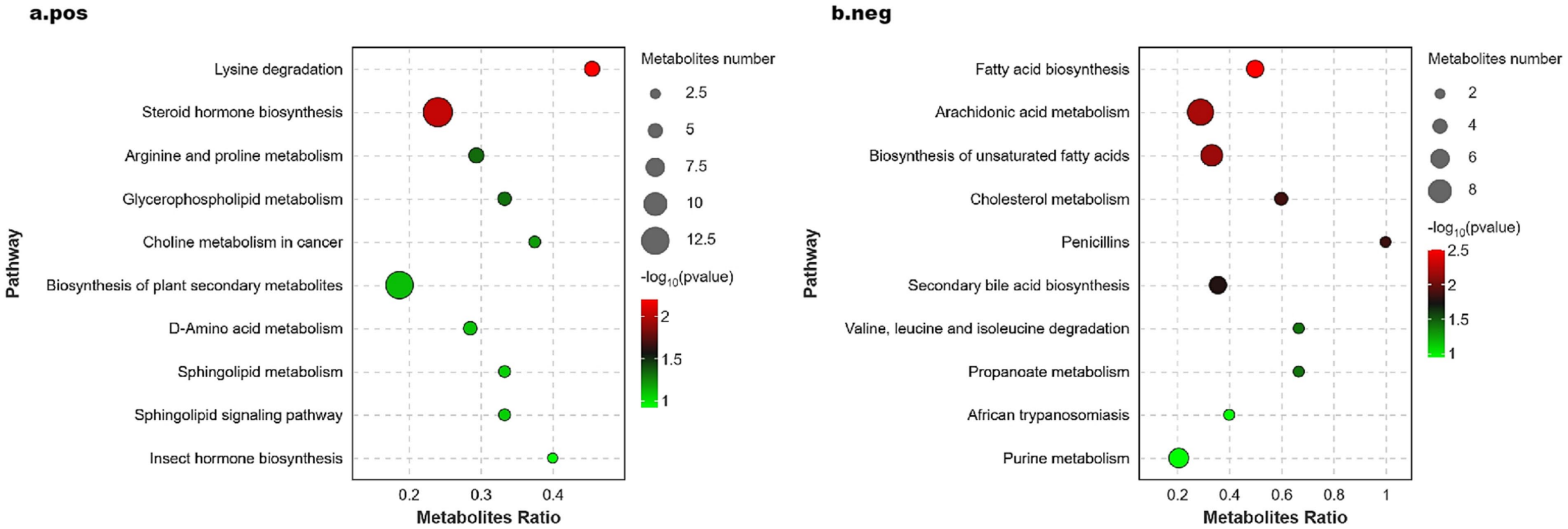
Significance bubble plot of metabolic pathways in positive **(A)** and negative **(B)** ion mode.

The KEGG Pathway function was used to annotate the differential metabolites, identifying 121 and 110 differential metabolites in the positive and negative ion modes, respectively. This information is illustrated in the significance bubble diagram, where a higher value of -log_10_ (*p*-value) indicates greater significance in enrichment. The size of the bubbles in the diagram corresponds to the number of differential metabolites associated with each pathway, with larger bubbles indicating a greater number of metabolites enriched in that pathway. As shown in Figure 5, the differential metabolites identified in the positive ion mode were enriched in several pathways (see Figure 5A), including lysine degradation, steroid hormone biosynthesis, arginine and proline metabolism, and glycerophospholipid metabolism, all of which are critical to amino acid and lipid metabolism. Conversely, the differential metabolites in the negative ion mode (see Figure 5B) showed enrichment in pathways related to fatty acid biosynthesis, arachidonic acid metabolism, biosynthesis of unsaturated fatty acids, cholesterol metabolism, and propanoate metabolism.

## 4 Discussion

In this study, we used gas chromatography–mass spectrometry (GC–MS) to examine the metabolomic profiles of intestinal microorganisms in Tibetan sheep across both cold and warm seasons. Our results revealed significant fluctuations in intestinal metabolites between these seasonal conditions. Notably, the concentrations of several metabolites, including cucurbitacin I, ursolic acid, gutta percha, and cholesterol sulfate, were substantially down-regulated in the intestinal metabolism of Tibetan sheep during the warm season compared to the cold season. The significant up-regulation of fatty acid metabolic pathways in the intestine of Tibetan sheep during the cold season compared with other highland ruminants (e.g., yak) is similar to the strategy employed by yak in coping with the low-temperature environment (11). This suggests that lipid metabolism is a common mechanism for energy reserves in highland animals. However, the unique up-regulation of secondary bile acid synthesis pathways in Tibetan sheep may be associated with their adaptation to roughage and differences in gut microbial composition, which differ from the metabolic profile of low-altitude sheep (10). Cucurbitacin I, a member of the cucurbitacin family, is a tetracyclic triterpenoid compound with a bitter taste and various physiological activities, including antioxidant, anticancer, and anti-inflammatory properties (12). Previous research has shown that cucurbitacin can influence the composition of gut microorganisms and mitigate intestinal inflammatory responses in animal models (13). Therefore, it is plausible that cucurbitacin I may exert anti-inflammatory or antioxidant effects in modulating intestinal microorganisms in Tibetan sheep during the cold season, aiding their adaptation to harsh low-temperature environments.

Ursolic acid, also known as ursolic acid, is a pentacyclic triterpenoid consisting of over 30 carbon atoms, characterized by a pentacyclic polyhydropinocampheus skeleton and classified as an *α*-coumarinic alkane type (α-amyrane). This compound exhibits a variety of biological functions, including anti-inflammatory (14, 15), antimicrobial (16, 17), anticancer (18–20), and antioxidant properties (21, 22, 36). Recent studies have suggested that ursolic acid can support intestinal homeostasis and overall health by influencing epithelial barrier function, intestinal mucin expression, bacterial flora composition, and immune cell activity (23–25). In our study, we noted a significant reduction in the content of ursolic acid was observed in the intestines of Tibetan sheep during the warm season compared to the cold season. This decrease may suggest that ursolic acid plays a protective role in maintaining intestinal mucosal barrier function and regulating the structure of intestinal flora under cold conditions, thereby enhancing the immune function of the organism.

Short-chain fatty acids (SCFAs) are a group of fatty acids that are crucial in the ruminant intestine, contributing to various biological functions such as regulating the ecological balance of intestinal microorganisms (26, 27, 37), providing energy to the host (28), and repairing the intestinal barrier (29). These functions are essential for maintaining intestinal health, preventing diseases, and promoting the overall well-being of the host. The observed decline in SCFA content in the intestinal tract of Tibetan sheep during the cold season may indicate a more favorable ecological balance and enhancing disease resistance within the intestinal flora of Tibetan sheep during the warm season.

The functional annotation of differential metabolites revealed that a significant proportion of these metabolites were categorized as fatty acids and their derivatives. This observation suggests that the differential metabolites identified in cold and warm seasons may be involved in fatty acid-related metabolic processes within the organism of Tibetan sheep. The KEGG functional analysis of the identified differential metabolites indicated that those upregulated during the cold season, particularly in negative ion mode, were predominantly enriched in metabolic pathways related to lipid compounds. These pathways include arachidonic acid metabolism, unsaturated fatty acid biosynthesis, cholesterol metabolism, and secondary bile acid biosynthesis, among others.

Fatty acids are a primary energy source for Tibetan sheep and play a crucial role in facilitating metabolism, growth, and development (30). The harsh environmental conditions during the cold season promote the catabolism of adipose tissue, resulting in elevated levels of fatty acids within intestinal metabolites. Consequently, during this period, when pasture availability is limited, Tibetan sheep depend on lipid metabolic pathways to maintain a stable energy supply.

Secondary bile acids (SBAs) are synthesized from primary bile acids through conjugation with glycine and taurine, forming bound primary bile acids (31). Upon entering the intestine, these compounds undergo hydrolysis and dehydroxylation, yielding SBAs that act as surface-active agents in the intestinal milieu. They aid in lipid emulsification, improve lipid digestion, and facilitate absorption by intestinal epithelial cells. Moreover, SBAs can influence energy metabolism by interacting with membrane and nuclear receptors, thereby regulating energy homeostasis. They may also modulate the expression of genes related to inflammatory responses, which helps maintain the host’s immune homeostasis (32, 33).

This research indicates that the levels of deoxycholic acid in the intestines of Tibetan sheep increase singnificantly during the cold season compared to the warm season. The increased concentration of deoxycholic acid in Tibetan sheep during colder months may modulate the biosynthesis pathway of secondary bile acids, thereby facilitating and improving the digestion and absorption of lipids and fat-soluble vitamins. This alteration in bile acid levels may also play a role in regulating the composition and metabolic functions of the gut microbiota in the host. Therefore, the upregulation of secondary bile acids in Tibetan sheep during the cold season may enhance fatty acid digestion, providing additional energy for the organism while also regulating inflammatory responses to support immune function.

In this study, we demonstrated that substantial alterations in cold-season secondary bile acids (e.g., deoxycholic acid) and SCFAs can potentially impact host behavior through the microbial-gut-brain axis. For instance, SCFAs have been observed to modulate feeding behavior and stress responses by stimulating intestinal chromaffin cells to release 5-hydroxytryptamine (5-HT), which is then transmitted to the central nervous system via the vagus nerve (34). Conversely, secondary bile acids may Secondary bile acids may regulate the activity of the hypothalamic–pituitary–adrenal axis (HPA axis) by binding to farnesoid X receptor (FXR) to reduce cortisol levels and enhance the host’s acclimatization to the cold environment (32).Together, these mechanisms support the maintenance of energy homeostasis and behavioral adaptation in Tibetan sheep during the cold season.

## 5 Strengths and limitations

The present study systematically revealed, for the first time, the differences in rectal metabolites and their functional pathways between the cold and warm seasons in Tibetan sheep, providing a new perspective on the mechanism of seasonal adaptation in plateau ruminants. However, the study is not without limitations. Firstly, the gut microbiome data were not combined to clarify the causal relationship between metabolite changes and specific flora. For instance, the synthesis of secondary bile acids may be driven by specific anaerobic bacteria (e.g., *Clostridium scindens*), which requires validation through the integration of metagenomics in future studies. Additionally, the limited sample size (*n* = 12) may compromise the statistical validity of the findings. Consequently, it is recommended that the sample size be expanded and that dynamic analysis be performed at multiple time points in subsequent studies.

Recent multi-omics studies have demonstrated that the integration of the metabolome and microbiome can provide a more comprehensive resolution of host–microbe interactions (35), which will be the focus of our subsequent research.

## 6 Conclusion

This study analyzed the metabolites of the rectal flora of Tibetan sheep across both cold and warm seasons, revealing significant differences in metabolite profiles between the two periods. The energy metabolism of Tibetan sheep increased during the cold season, characterized by elevated levels of fatty acids and their derivatives within metabolic pathways. The higher concentrations of fatty acids in intestinal metabolites supported energy provision in the cold environment and helped alleviatd oxidative stress, thereby mitigating the adverse effects of cold stress. At the same time, metabolites such as short-chain fatty acids and bile acids in the gut can influence the host’s emotional state and feeding behavior, among others, through a complex bidirectional regulatory system, the microbe-gut-brain axis. Consequently, this research provides theoretical insights into the mechanisms underlying nutritional stress during the cold season, the impact of cold stress, and the regulation of the “microbe-gut-brain axis” in Tibetan sheep.

## Data Availability

The original contributions presented in the study are publicly available. These data can be found in repository: OMIX database of NGDC. The OMIX accession number is: OMIX009476.
